# Kahweol Ameliorates the Liver Inflammation through the Inhibition of NF-κB and STAT3 Activation in Primary Kupffer Cells and Primary Hepatocytes

**DOI:** 10.3390/nu10070863

**Published:** 2018-07-04

**Authors:** Hye-Young Seo, Mi-Kyung Kim, So-Hee Lee, Jae Seok Hwang, Keun-Gyu Park, Byoung Kuk Jang

**Affiliations:** 1 Department of Internal Medicine, Keimyung University School of Medicine, Daegu 42601, Korea; seo568@hanmail.net (H.-Y.S.); mdkmk@dsmc.or.kr (M.-K.K.); jy16162727@naver.com (S.-H.L.); gastro@dsmc.or.kr (J.S.H.); 2 Institute for Medical Science, Keimyung University School of Medicine, Daegu 42601, Korea; 3 Department of Internal Medicine, School of Medicine, Kyungpook National University, Daegu 41944, Korea; kpark@knu.ac.kr

**Keywords:** liver inflammation, lipopolysaccharide, kahweol, primary hepatocyte, primary Kupffer cells

## Abstract

Gut derived bacterial endotoxins, such as lipopolysaccharide (LPS), are involved in one of the important mechanisms that lead to inflammation associated with various liver diseases, including nonalcoholic fatty liver disease and alcoholic liver disease. Kahweol is a coffee-specific diterpene present in coffee bean and exhibits anti-angiogenic and anti-inflammatory activities. However, to date, the effect of kahweol on liver inflammation remains unknown. In this study, we examined whether kahweol exhibits a protective effect by inhibiting liver inflammation in primary Kupffer cells and primary hepatocytes cultures as well as their co-cultures. Kahweol decreased the LPS-induced production of interleukin 1 alpha, interleukin 1 beta, interleukin 6, and tumor necrosis factor alpha. The inhibitory effect of kahweol on the liver inflammation was associated with the down regulation of LPS-stimulated phospho-nuclear factor kappa B and -signal transducer and activator of transcription 3 expression. These results suggest that kahweol might be a novel potent agent to treat liver inflammation induced by LPS.

## 1. Introduction

Lipopolysaccharide (LPS), an endotoxin, is present in the outer membrane of gram-negative bacteria. The induced toxicity is characterized by injury to various organs including the liver, kidney, and brain [1,2]. Intestinal LPS plays an important role in the progression of various liver diseases including nonalcoholic fatty liver disease (NAFLD) and alcoholic liver disease (ALD) [3]. An increase in LPS levels in the liver causes hepatocyte damage and stimulates hepatic macrophages [4,5]. This leads to the release of pro-inflammatory cytokines such as interleukin (IL)-1, IL-6, and tumor necrosis factor-α (TNF-α). The secretion of these inflammatory cytokines leads to chronic inflammation, which in turn causes hepatitis, fibrosis, and cirrhosis [6,7,8,9].

Several signaling pathways are involved in the LPS-induced inflammatory response of the liver. One of these mechanisms involves nuclear factor kappa B (NFκB), a major downstream transcription factor that initiates the transcription of inflammatory mediator genes during the induction of inflammatory stimuli by molecules such as LPS [10,11]. Signal transducer and activator of transcription 3 (STAT3) is another important transcriptional factor involved in the immune response and inflammation [12]. Therefore, targeting NFκB and STAT3 signaling pathways is considered as an attractive therapeutic strategy for the development of anti-inflammatory drugs.

Coffee is one of the most popular beverages worldwide and its consumption is commonly known to be beneficial to human health [13]. Moreover, coffee is a mixture of many bioactive compounds including caffeine, diterpenes (kahweol and cafestol), polyphenols (ex. chlorogenic acids), and melanoidines [14] that were recently associated with the decreased risk of mortality [15], cardiovascular disease [16], hypertension [17], diabetes [18], and cancer [19]. Kahweol is a coffee-specific diterpene that is found in coffee beans and is present in the unfiltered coffee beverage [20]. Moreover, it is well-known that kahweol exhibits anti-carcinogenic, anti-inflammatory, and anti-tumor progression properties [21,22]. According to several studies, coffee consumption reduces the risk of advanced liver diseases as well as hepatocellular carcinoma [23]. Recently, a study reported that kahweol inhibits hepatic fibrosis in vitro and in vivo [24]. Therefore, in this study, we evaluated the anti-fibrosis and anti-inflammatory effects of kahweol on liver cells.

## 2. Materials and Methods 

### 2.1. Reagents and Chemical

Kahweol was purchased from LKT Laboratories Inc. (St. Paul, MN, USA), lipopolysaccharide (LPS, *Escherichia coli* 055; B5) was purchased from Sigma Aldrich (St. Louis, MO, USA). Anti-phospho-NFκB antibody, anti-phospho-ERK (Th202/Tyr204) antibody, anti-phospho-JNK (Thr182/Tyr185) antibody, anti-phospho-STAT3 (Tyr705) and anti-GAPDH antibody were purchased from Cell Signaling Technology (Beverly, MA, USA).

### 2.2. Isolation of Primary Kupffer Cells (KC) and Primary Hepatocytes (HC)

C57BL/6 HC and KC were isolated by perfusing the liver via the portal vein. The liver was perfused with resuspension buffer (5.4 mmol/L KCl, 0.44 mmol/L KH_2_PO_4_, 140 mmol/L NaCl, 0.34 mmol/L Na_2_HPO_4_, 0.5 mmol/L EGTA, 25 mmol/L Tricine, pH 7.2) at 5 mL/min for 10 min and then perfused with collagenase buffer (collagenase type I (Worthington Biochemical Corp, Freehold, NJ, USA) 0.75 mg/mL, Ca^2+^ and Mg^2+^ free Hanks Balanced salt solution (HBSS)) at 5 mL/min for 10 min. After perfusion, the liver was shaken for 20 min at 37 °C and filtered through a 70 µm nylon mesh and centrifugation at 42× *g* for 5 min at 4 °C. Supernatants were collected for KC isolation, and the HC pellets were re-suspended in serum-free Williams’ medium E (WEM, Sigma). HC were plated in 10% fetal bovine serum Williams’ medium E on type I collagen-coated dishes (IWAKI Scitech Kiv, Tokyo, Japan). The viability of HC were always greater than 85%. After a 2- to 3-h incubation, the medium was exchanged with medium 199 (SIGMA, St. Louis, MO, USA). For the isolation of KC, supernatants was centrifuged at 562× *g* for 10 min and then the KC pellets subjected to OptiPrep^TM^ (SIGMA, St. Louis, MO, USA) density-gradient centrifugation. The cells were washed with HBSS, and plated in 10% fetal bovine serum RPMI 1640 medium (GIBCO-BRL, Grand Island, NY, USA). After 10–20 min of incubation, unattached cells were washed away to obtain purified KC. Cells purity were confirmed by F4/80 mRNA levels (Figure S1). HC and KC were treated with chemicals in 0.5% FBS with or without LPS (10 ng/mL), and then subsequently processed for isolation of protein and RNA as described below.

### 2.3. Plating of Primary KC and Primary HC Co-Cultures

Primary KC and primary HC were plated at a 1:4 (KC:HC) ratio onto 6-well type I collagen-coated plate with Medium 199 or Williams’ medium E. Co-cultures were treated with chemicals in 0.5% FBS with or without LPS (10 ng/mL), and then subsequently processed for isolation of protein and RNA as described below.

### 2.4. Quantitative Real-Time (qRT)-RCR 

Total RNA was isolated from cells, using the Trizol reagent (Invitrogen, MA, USA). Reverse transcription was performed using the Maxima First Strand cDNA synthesis kit (Thermo scientific, Waltham, MA, USA). Quantitative real-time RT-PCR was performed using a SYBR Green PCR master mix kit (Roche Diagnostics, Indianapolis, IN, USA) and a Light Cycler 96 instrument (Roche Diagnostics, Indianapolis, IN, USA). PCR parameters were as follows: 45 cycles of 95 °C for 30 s, 60 °C for 10 s, and 72 °C for 15 s. Primer sequences were as follows: mouse IL1α, 5′-CAACGTCAAGCAACGGGAAG-3′, and reverse, 5′-AAGGTGCTGATCTGGCTTGG-3′; mouse IL1β forward, 5′-CTTTCCCGTGGACCTTCCAG-3′, and reverse, 5′-AATGGGAACGTCACACACCA-3′; mouse IL6 forward, 5′-TTGCCTTCTTGGGACTGATG-3′, and reverse, 5′-CTCATTTCCACGATTTCCCA-3′; mouse TNFα forward, 5′-ACCGTCAGCCGATTTGCTAT-3′, and reverse, 5′-CCGGACTCCGCAAAGTCTAA-3′; mouse GAPDH forward, 5′-ACGACCCCTTCATTGACCTC-3′, and reverse, 5′-ATGATGACCCTTTTGGCTCC-3′. GAPDH were used as an internal standard. 

### 2.5. Determination of Inflammatory Cytokine Levels 

The cells seeded in six-well plates and the treated with kahweol and LPS. Supernatant fractions harvested and centrifuged at 1000 rpm for 5 min and conditioned media (CM) were collected and stored at −80 °C. Levels of IL1α, IL1β, IL6, and TNFα were detected via ELISA kit according to the manufacturer’s instructions (R&D Systems, Abingdon, UK). 

### 2.6. Western Blot Analysis

Total protein was extracted via RIPA buffer (Thermo scientific, Waltham, MA, USA), and the concentration measured by BCA protein assay (Thermo scientific, Waltham, MA, USA) according to the manufacturer’s protocols. Cell lysates of 10 μg were separated by SDS-PAGE and then transferred onto PVDF (Millipore, Billerica, MA, USA) membrane, which was blocked for 1 h at room temperature with 5% Skim milk in Tris-buffered saline containing 0.1% Tween 20. The membrane was incubated with primary antibody and appropriate horseradish peroxidase-conjugated secondary antibody, and then developed using the Clarity™ Western ECL substrate kit (Bio-Rad, Richmond, CA, USA). Signal intensities were quantitated by densitometry using the ImageJ software (NIH, Bethesda, MD, USA). 

### 2.7. Statistical Analysis 

Data are expressed as means ± SEM. ANOVA was used to determine significant differences in multiple comparisons and was performed by the Duncan test. Values of *p* < 0.05 were considered statistically significant.

## 3. Results

### 3.1. Kahweol Inhibited LPS-Stimulated Inflammatory Cytokine Levels in Primary KC and Primary HC

**Figure 1:**
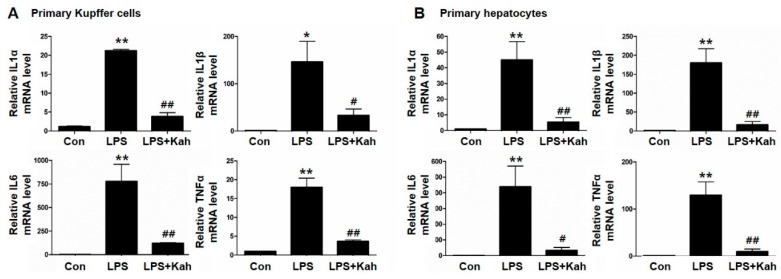
Effect of kahweol on lipopolysaccharide (LPS)-induced inflammatory cytokine mRNA expression in primary Kupffer cells (KC) and primary hepatocyte (HC). (**A**,**B**) Representative real-time RT-PCR analysis of IL1α, IL1β, IL6, and TNFα mRNA expression in primary KC (**A**) and primary HC (**B**). Data in the bar graph are mean ± SEM of three independent measurements. * *p* < 0.05, ** *p* < 0.01 compared with control (Con), ^#^ *p* < 0.05, ^##^ *p* < 0.01 compared with LPS. Kah, Kahweol.

**Figure 2:**
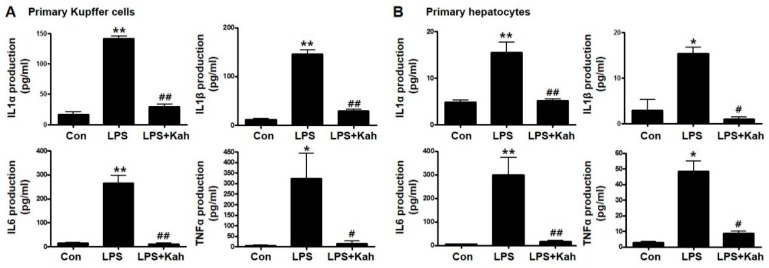
Effect of kahweol on lipopolysaccharide (LPS)-induced production of IL1α, IL1β, IL6, and TNFα. Primary Kupffer cell (KC) (**A**) and primary hepatocyte (HC) (**B**) media were collected after LPS and kahweol (kah) treatment, the production of IL1α, IL1β, IL6, and TNFα were measured using the ELISA kits. Data in the bar graph are mean ± SEM of three independent measurements. * *p* < 0.05, ** *p* < 0.01 compared with control (Con), ^#^ *p* < 0.05, ^##^ *p* < 0.01 compared with LPS. Kah, Kahweol.

The inflammatory cytokines IL1α, IL1β, IL6, and TNFα play an important role during the hepatic inflammation [25,26]. We examined the effects of kahweol on the mRNA expression of IL1α, IL1β, IL6 and TNFα. For this purpose, we isolated primary KC and primary HC from mice livers and used these cells in our experiments. LPS effectively increased the mRNA expression and protein secretion of the inflammatory cytokines (Figure 1 and Figure 2). However, these increased levels were markedly reduced with kahweol treatment. The anti-inflammatory effect of kahweol was confirmed in primary KC as well as primary HC.

### 3.2. Kahweol Inhibited Inflammatory Cytokine Levels in Primary KC and Primary HC Co-Cultures

**Figure 3:**
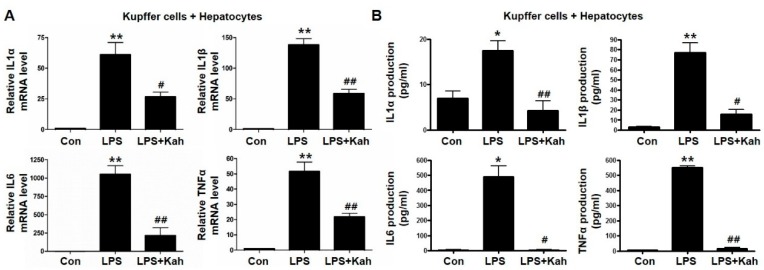
Effect of kahweol on lipopolysaccharide (LPS)-induced expression and production of IL1α, IL1β, IL6, and TNFα in primary Kupffer cell (KC) and primary hepatocyte (HC) co-culture. (**A**) Representative real-time RT-PCR analysis of IL1α, IL1β, IL6, and TNFα mRNA expression in co-culture of primary KC with primary HC. Data in the bar graph are mean ± SEM of three independent measurements. ** *p* < 0.01 compared with control (Con), ^#^ *p* < 0.05, ^##^ *p* < 0.01 compared with LPS. (**B**) Primary KC and primary HC co-cultures media were collected after LPS and kahweol (kah) treatment, the production of IL1α, IL1β, IL6, and TNFα were measured using the ELISA kits. Data in the bar graph are mean ± SEM of three independent measurements. * *p* < 0.05, ** *p* < 0.01 compared with control (Con), ^#^ *p* < 0.05, ^##^ *p* < 0.01 compared with LPS.

The activation of KC plays an important role in the pathogenesis of inflammation [27,28]. Moreover, the cellular interaction between KC and HC is triggered by the production and secretion of inflammatory cytokines. Further, we used primary KC and primary HC co-culture as an in vitro model of inflammation. Inflammatory cytokine levels were increased in primary KC and primary HC co-cultures compared to primary HC cultures. Kahweol inhibited the LPS-stimulated mRNA expression and protein secretion of inflammatory cytokines in primary KC and primary HC co-cultures (Figure 3A,B).

### 3.3. Kahweol Inhibited the NF-κB Activation

**Figure 4:**
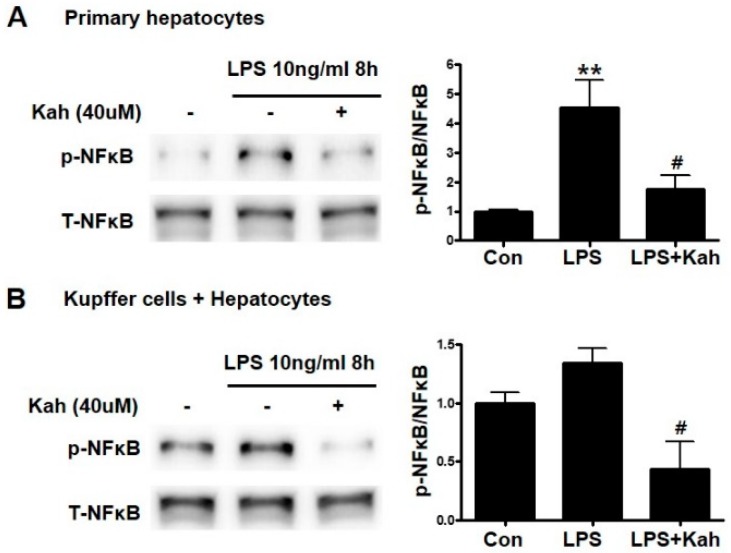
Effect of kahweol on lipopolysaccharide (LPS)-induced activation of the NFκB pathway. (**A**) Primary hepatocytes were pretreated with kahweol (Kah) and then stimulated with or without LPS. The expression of phosphor (p)-NFκB was analyzed by western blot. Data represented in the bar graph are the mean ± SEM of three independent measurements. ** *p* < 0.01 compared with control (Con), ^#^ *p* < 0.01 compared with LPS. (**B**) Co-culture of Kupffer cells with hepatocytes were pretreated with the kahweol and then stimulated with or without LPS. The expression of phosphor (p)-NFκB were analyzed by western blot. Data represented in the bar graph are the mean ± SEM of three independent measurements. ^#^ *p* < 0.01 compared with LPS. T-NFκB, total NFκB.

NFκB signaling pathway was reported to be important in the LPS induced liver inflammation [29]. Therefore, we investigated the effects of kahweol on the NFκB signaling pathways. The phosphorylation of NFκB was higher in primary HC treated using LPS; however, kahweol decreased phosphorylated NFκB level (Figure 4A). In primary KC and primary HC co-cultures, the NFκB phosphorylation tended to increase slightly owing to LPS-stimulation; however, it was significantly reduced by kahweol treatment (Figure 4B).

### 3.4. Involvement of STAT3 and Mitogen-Activated Protein Kinase (MAPK) Activation in the Inhibitory Effect of Kahweol on Inflammation

**Figure 5:**
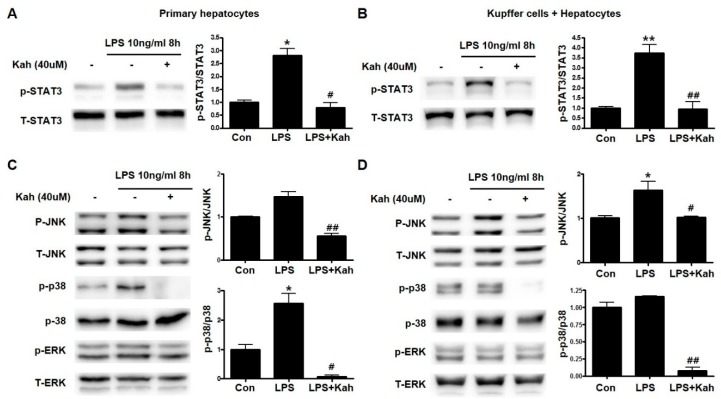
Effect of kahweol on lipopolysaccharide (LPS)-induced activation of the STAT3 and MAPK signaling pathways. (**A**,**B**) Primary hepatocytes (**A**) and co-culture of primary Kupffer cells with hepatocytes (**B**) were pretreated with kahweol (Kah) and were pretreated with kahweol and then stimulated with or without LPS. The expression of phospho (p)-STAT3 was analyzed by western blot. Data represented in the bar graph are the mean ± SEM of three independent measurements. * *p* < 0.05, ** *p* < 0.01 compared with control (Con), ^#^ *p* < 0.05, ^##^ *p* < 0.01 compared with LPS. T-STAT3, total STAT3 (**C**,**D**) Primary hepatocytes (**C**) and co-culture of primary Kupffer cells with hepatocytes (**D**) were pretreated with kahweol and then stimulated with or without LPS. The expression of p-JNK, p-p38, and p-ERK were analyzed by western blot. Data represented in the bar graph are the mean ± SEM of three independent measurements. * *p* < 0.05 compared with control (Con), ^#^ *p*< 0.05, ^##^ *p* < 0.01 compared with LPS. T- JNK, total JNK. T-ERK, total ERK.

STAT3 and MAPK are crucial mediators during the production of inflammatory cytokines and are reported to induce NFκB activation [12]. Therefore, we examined the effect of kahweol on the STAT3 and MAPK activation. LPS increased phospho-STAT3 expression and kahweol significantly decreased this elevated phospho-STAT3 expression (Figure 5A,B). Similarly, we found that kahweol inhibited phospho-JNK and phospho-p38 MPAK but not phospho-ERK expression (Figure 5C,D). These results clearly demonstrated that kahweol inhibited the activation of STAT3 and MAPK to regulate the production of inflammatory cytokines. 

### 3.5. Kahweol Inhibited Primary HC Inflammation Induced by the Conditioned Media Obtained from LPS Treated Primary KC

**Figure 6:**
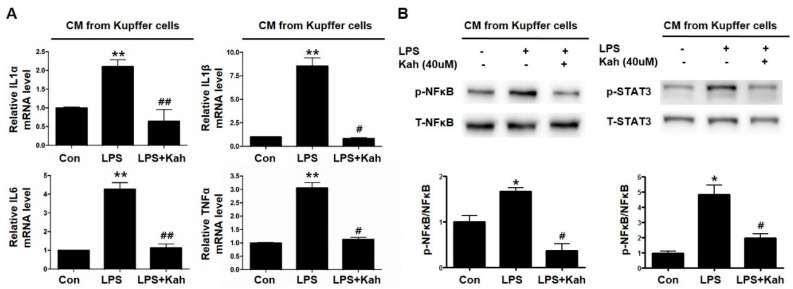
Kahweol inhibited primary HC inflammation induced by conditioned media in primary KC treated with LPS. (**A**) Representative real time RT-PCR analysis of the expression levels of IL1α, IL1β, IL6, and TNFα in primary HC treated with conditioned media (CM) from LPS and kahweol treated primary KC. ** *p* < 0.01 compared with control (con), ^#^ *p* < 0.05, ^##^ *p* < 0.01 compared with LPS. (**B**) Representative western blot of phospho (p)-NFκB and phospho (p)-STAT3 expression in primary HC treated with CM form LPS and kahweol treated primary KC. Data represented in the bar graph are the mean ± SEM of three independent measurements. * *p* < 0.05 compared with control (Con), ^#^ *p* < 0.05 compared with LPS. T- NFκB, total NFκB, T-STAT3, total STAT3.

Activated KC are a major source of inflammatory mediators including cytokines during inflammation in the liver [30]. Further, we examined whether the treatment of KC using kahweol could inhibit the hepatocyte inflammation. The mRNA levels of IL1α, IL1β, IL6, and TNFα were increased in primary HC incubated in the conditioned media obtained from LPS-treated primary KC. These elevated cytokine levels in primary HC were markedly inhibited by using the conditioned media obtained from kahweol-treated primary KC (Figure 6A). Moreover, in these experiments using the conditioned media from primary KC, the LPS-induced phospho-NFκB and phospho-STAT3 protein expressions were reduced by kahweol (Figure 6B). These results indicated that primary KC increase the inflammation of primary HC by the secretion of pro-inflammatory cytokines including IL1α, IL1β, IL6, and TNFα and kahweol blocks the secretion of such inflammatory cytokines. 

## 4. Discussion

Our results demonstrate that kahweol suppresses the LPS-induced production of IL-1α, IL-1β, IL-6, and TNF-α in primary KC, primary HC, and their co-cultures. We found that the inhibitory effect of kahweol on the liver inflammation is associated with the downregulation of LPS-stimulated phospho-NFκB and -STAT3 expression.

Systemic and portal endotoxemia are important to determine the occurrence and progression of chronic liver diseases including ALD and NAFLD. Several previous studies reported that alcohol feeding induces endotoxemia in animal models [31] and that endotoxin levels in the plasma are significantly higher in patients with ALD than that in normal individuals [32,33]. Moreover, it was reported that the levels of portal endotoxins were increased in diet-induced nonalcoholic steatohepatitis animal models [34]. Endotoxemia induced by these aforementioned causes activates KCs and these activated KCs secrete various inflammatory cytokines, chemokines, and reactive oxygen species (ROS) [35,36].

Several in vitro studies revealed the anti-inflammatory effects of kahweol that are associated with the inhibition of cyclooxygenase-2 (COX2) and inducible nitric oxide synthase (iNOS). Moreover, kahweol is reported to target NFκB/STAT1 in macrophage cells [37,38,39]. However, this protective effect was mainly studied in RAW264.7 cells. Therefore, we isolated primary KC and primary HC in order to evaluate the inhibitory effect of kahweol on the liver inflammation. In this study, kahweol significantly decreased the LPS-stimulated levels of pro-inflammatory cytokines in primary KC and primary HC.

STAT3 and MAPK were reported to play an important role in the regulation of LPS-induced inflammation by NFκB activation [10,30]. It was reported that kahweol induces apoptosis in cancer cells by decreasing the STAT3 levels and in turn inhibits hepatic fibrosis in the liver [24,40]. Similarly, our results confirmed that kahweol reduces the liver inflammation by decreasing the LPS-induced STAT3 expression. Additionally, kahweol exhibited inhibitory effects on phospho-JNK and phospho-p38 MAPK in primary KC and primary HC. Therefore, a decrease in the expression of IL-1α, IL-1β, IL-6, and TNF-α owing to the inhibitory effect of kahweol might be associated with the blockade of these genes.

The pathogenesis of liver injury requires interactions among HCs, endothelial cells, and KCs. The number of HCs is a limiting factor in the study of complex interactions among multiple cytokines and cell types that play a role in vivo in inflammatory disease. To overcome this limitation, attempts were made to establish a co-culture model by integrating the components of the immune system such as HCs and KCs. The HC and KC co-culture model is used to investigate the effect of secondary cytokines on HC via the stimulation of KC-mediated cytokine secretion [28,41,42]. In this study, kahweol significantly decreased the LPS-induced pro-inflammatory cytokine levels in primary KC and primary HC co-culture model. Moreover, KCs release ROS, nitric oxide, cytokines, and chemokines that modulate HC and non-parenchymal cell death [43]. Therefore, we examined the effect of cytokines secreted by KC on HC. As a result, the inflammatory response of HC induced by the conditioned media of LPS-stimulated KCs was prevented by kahweol treatment.

Several studies demonstrated that nuclear erythroid-derived 2-related factor (NRF2) contributes to the anti-inflammatory process [44,45]. NRF2 pathway is considered as one of the therapeutic targets in various liver diseases. Multiple stimuli such as viruses and chemical drugs activate NRF2 and subsequently it gets translocated into the nucleus. In the nucleus, Nrf2 binds to antioxidant response element -target genes such as heme oxygenase 1 and nicotinamide adenine dinucleotide phosphate: quinone oxidoreductase 1 [46]. Previously, kahweol was reported to increase NRF2 expression [47]. In our study, the protein expression of HO-1, a target gene of NRF2, was increased during kahweol treatment (Figure S2). Additionally, the inhibition of NRF2 expression using siRNA inhibited HO-1 expression. However, the inhibitory effect of kahweol on inflammatory cytokines was prominent despite the inhibition of NRF2 expression using siRNA. These results suggest that the anti-inflammatory effect of kahweol might be associated with other mechanisms besides the NRF2 pathway. In this study, although we reported the aforementioned result, we observed a limitation indicating that the expression of NRF2 and HO-1 occurred in whole cell lysates. Biswas C et al. [48] reported that unlike cytoplasmic HO-1, nuclear HO-1 interacts with NRF2 and this complex exhibits cytoprotection. Therefore, further studies are required to determine the mediators through which kahweol plays an important role in liver disease treatment. 

## 5. Conclusions

In conclusion, our results demonstrate that kahweol significantly decreased LPS-stimulated inflammatory cytokines. This reduction was associated with the downregulation of NFκB, STAT3 and p38 MAPK. Therefore, kahweol might be a potential novel agent to treat the liver inflammation.

## Supplementary Materials

The following are available online at http://www.mdpi.com/2072-6643/10/7/863/s1, Figure S1: Comparison of F4/80 mRNA expression in primary HC and primary KC. The expression of F480 mRNA level was analyzed by real-time RT-PCR analysis. * *p* < 0.01 compared with primary HC, ** *p* < 0.01 compared with HC:KC, Figure S2: Anti-inflammatory effect of kahweol is independent of the NRF2 pathway. (A,B) Primary hepatocytes (HC) (A) and co-culture of primary Kupffer cells with hepatocytes (B) were pretreated with the kahweol and then stimulated with or without LPS. The expression of HO-1 were analyzed by western blot. Data represented in the bar graph are the mean ± SEM of three independent measurements. ^##^ *p* < 0.01 compared with LPS. (C) Primary HC transfected with NRF2 siRNA or control (Con)-siRNA for 48 h were subjected to real-time RT-PCR analysis using primers specific for NRF2, HO-1, IL1α and IL1β.C, control. L, LPS. L+K, LPS+Kahweol.
