# Supplementary material for: Kahweol Ameliorates the Liver Inflammation through the Inhibition of NF-κB and STAT3 Activation in Primary Kupffer Cells and Primary Hepatocytes
Source: Nutrients. 2018 Jul 4;10(7):863. doi: 10.3390/nu10070863 (PMC6073512; doi:10.3390/nu10070863)
Supplement: Supplementary file 1 [file nutrients-10-00863-s001.pdf]

Supplementary material and methods.

-siRNA for NRF2

Pre-designed siRNAs were purchased for NRF2 (Santa Cruz, Dallas, TX, USA)). Cells were transfected with 100 nM siRNA using the Lipofectamine RNAiMAX reagent (Invitrogen, Carlsbad, CA, USA) according to the manufacturer`s instructions. The effects of NRF2 siRNA on the expression of endogenous NRF2 confirmed by real-time RT-PCR.

- Quantitative real-time (qRT)-RCR

PCR parameters were as follows: 45 cycles of 95 °C for 30 s, 60 °C for 10 s, and 72 °C for 15 s. Primer sequences were as follows: mouse NRF2 forward, 5`-AAAGCACAGCCAGCACATTC-3`, and reverse, 5`-AATGGGGCTTTTTGATGACC-3`; mouse HO1 forward, 5`-TACCTTCCCGAACATCGACA-3`, and reverse, 5`-TCTGCAGGGGCAGTATCTTG-3`; mouse F4/80 forward, 5`-CTGAACATGCAACCTGCCAC-3`, and reverse, 5`-AGGTGGGACCACAGAGAGTT-3`. GAPDH was used as an internal standard

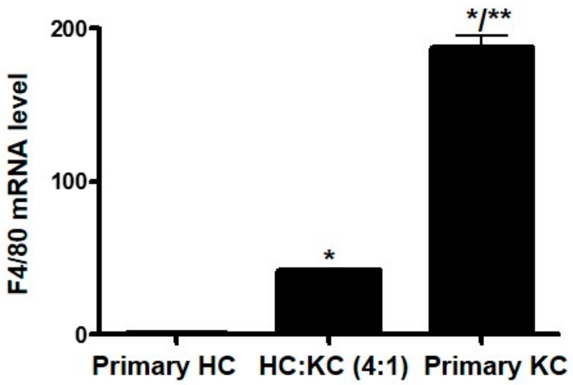

Figure S1. Comparison of F4/80 mRNA expression in primary HC and primary KC. The expression of F480 mRNA level was analyzed by real-time RT-PCR analysis. *P< 0.01 compared with primary HC, ** P< 0.01 compared with HC:KC.

Supplementary Figure 2

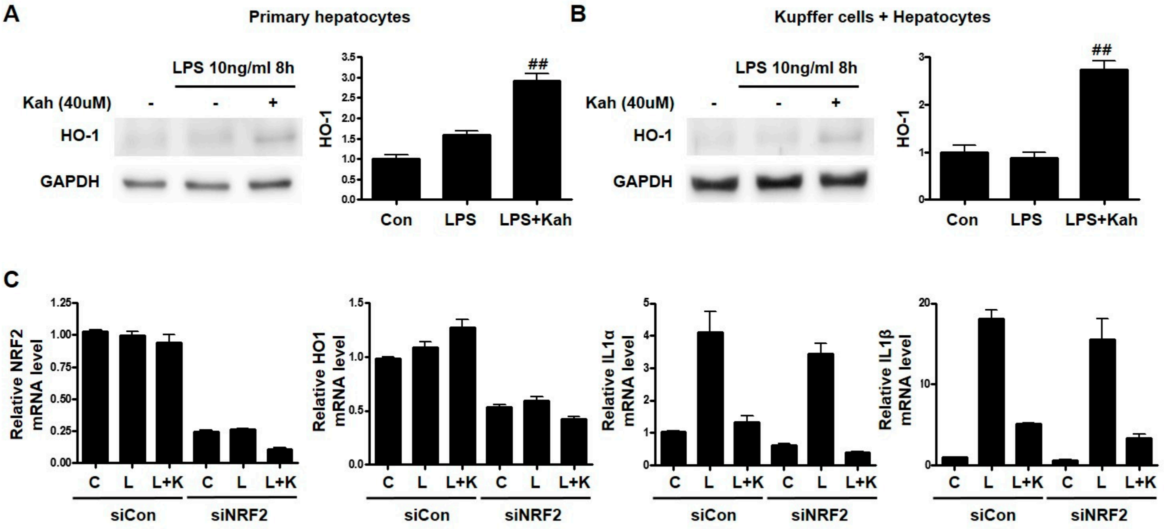

Figure S2. Anti-inflammatory effect of kahweol is independent of the NRF2 pathway. (A,B) Primary hepatocytes (HC) (A) and co-culture of primary kupffer cells with hepatocytes (B) were pretreated with the kahweol and then stimulated with or without LPS. The expression of HO-1 were analyzed by western blot. Data represented in the bar graph are the mean ± SEM of three independent measurements. $^{##}P< 0.01$ compared with LPS. (C) Primary HC transfected with NRF2 siRNA or control (Con)-siRNA for 48 h were subjected to real-time RT-PCR analysis using primers specific for NRF2, HO-1, IL1α and IL1β.C, control. L, LPS. L+K, LPS+Kahweol.
